# P-1682. Evaluation of Antibiotic Prescribing Practices for Pediatric Acute Otitis Media and Community-Acquired Pneumonia in an Emergency Department

**DOI:** 10.1093/ofid/ofae631.1848

**Published:** 2025-01-29

**Authors:** Dara S Petel, Leo Cheong, Gregory Harvey, Olivia Ostrow, Kathryn E Timberlake, Michelle Science

**Affiliations:** The Hospital for Sick Children, Toronto, ON, Canada; The Hospital for Sick Children, Toronto, ON, Canada; The Hospital for Sick Children, Toronto, ON, Canada; Division of Pediatric Emergency Medicine, The Hospital for Sick Children, University of Toronto, Toronto, Ontario, Canada; The Hospital for Sick Children, Toronto, ON, Canada; The Hospital for Sick Children, Toronto, ON, Canada

## Abstract

**Background:**

Acute otitis media (AOM) and community-acquired pneumonia (CAP) are common reasons for antibiotic prescriptions in children. As a result, antimicrobial stewardship efforts ensuring evidence-informed antibiotic prescriptions may be impactful. The objective of this study was to assess whether antibiotic prescriptions for AOM and CAP in a pediatric emergency department (ED) are consistent with current guidelines, and to identify opportunities for improvement.

Duration of antibiotics prescribed for acute otitis media

**Figure:**
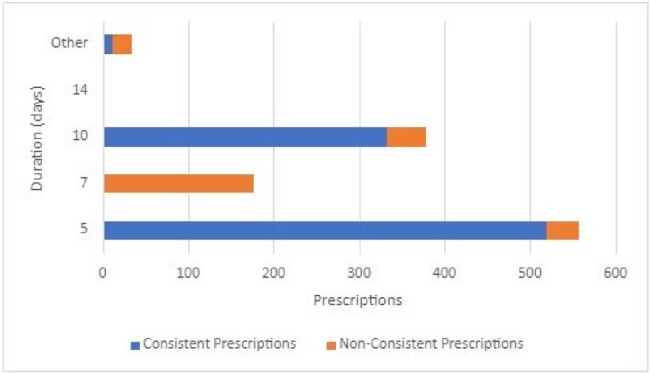

Bar graph depicting prescribed durations of therapy for acute otitis media, Prescriptions consistent with current guideline recommendations are in blue, while those that aren’t are in orange. Guidelines advise 10 days, rather than 5 days, of antibiotic therapy for children <2, perforated otitis media, treatment failure and recurrent otitis media.

**Methods:**

We conducted a retrospective review of outpatient antibiotic prescriptions for AOM and CAP in the ED of a pediatric hospital from September 2022 to September 2023. Patients ages 0 – 18 years discharged from the ED with a diagnosis of AOM or CAP were identified using the electronic medical record system. Exclusion criteria included absence of a new antibiotic prescription, concomitant infections requiring antibiotic treatment, hospital admission, and patients with immunocompromising conditions or medications. Prescriptions were considered consistent with guidelines if they followed the Canadian Paediatric Society recommendations. Descriptive statistics were used for analysis.

Duration of antibiotics prescribed for community-acquired pneumonia

**Figure:**
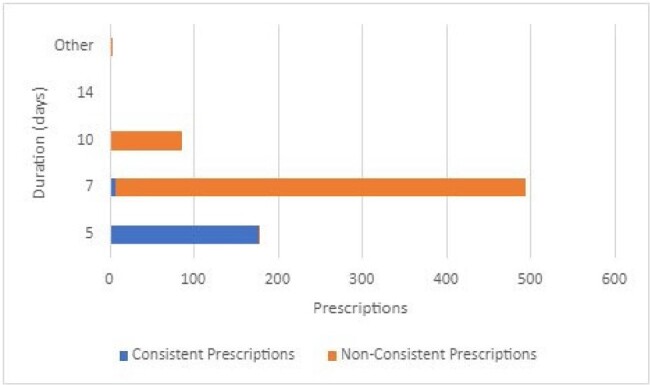

Bar graph depicting prescribed durations of therapy for community-acquired pneumonia. Prescriptions consistent with current guideline recommendations are in blue, while those that aren’t are in orange.

**Results:**

A total of 1143 and 765 cases of AOM and CAP were included, respectively. Of the prescriptions for AOM, 688 (60%) were consistent with current guidelines. The 454 prescriptions that were not guideline-consistent were due to duration (n=281, 62%, Figure 1), dosing interval (n=142, 31%), antibiotic selection (n=74, 16%), and dose (n=38, 8.4%). Deferred prescriptions were provided to 177 (16%) patients; an additional 139 (12%) were eligible, but prescribed treatment. For CAP, 146 (19%) prescriptions were consistent with current guidelines. Of the 618 prescriptions that were not consistent, the majority were due to duration (n=576, 92%, Figure 2), dosing interval (n=134, 22%), antibiotic selection (n=38, 6%) and dose (n=14, 2%).

**Conclusion:**

A significant number of patients with AOM (40%) and CAP (79%) were given antibiotic prescriptions that were not consistent with current guideline recommendations, with the most common reason being prolonged duration. This identifies an important antimicrobial stewardship opportunity in the ED and likely other outpatient settings.

**Disclosures:**

**Kathryn E. Timberlake, PharmD**, Avir Pharma: Advisor/Consultant|Sanofi: Honoraria|Wolters Kluwer: Advisor/Consultant

